# Florfenicol and Florfenicol Amine Quantification in Bull Serum and Seminal Plasma by a Single Validated UHPLC-MS/MS Method

**DOI:** 10.1155/2023/6692920

**Published:** 2023-05-27

**Authors:** Anisa Bardhi, Juan E. Romano, Giampiero Pagliuca, Alice Caneschi, Andrea Barbarossa

**Affiliations:** ^1^Department of Veterinary Medical Sciences, University of Bologna, Ozzano Emilia (BO), Italy; ^2^3R Ranch, Somerville, TX, USA; ^3^Cooperative Agriculture Research Center, College of Agriculture and Human Sciences of Prairie View A&M University, Prairie View, TX, USA; ^4^Health Sciences and Technologies-Interdepartmental Centre for Industrial Research (CIRI-SDV), University of Bologna, Ozzano dell'Emilia (BO), Italy

## Abstract

Florfenicol is a broad-spectrum antibiotic belonging to the amphenicols class that inhibits protein synthesis by binding to bacteria's ribosomal subunits. This drug is commonly used in veterinary medicine to treat bacterial infectious diseases in cattle, swine, poultry, and fish. The proposed method uses a quick protein precipitation with acetonitrile for the extraction of florfenicol and florfenicol amine in serum and seminal plasma, followed by analysis in UHPLC-MS/MS for their simultaneous quantification. A BEH C18 reversed-phase column was chosen for analyte separation, allowing to obtaining sharp and symmetrical peak shapes in a chromatographic run of just 3.5 min under programmed conditions. Two specific transitions were observed for each analyte, and florfenicol-d3 was used as the internal standard. The approach was fully validated in each matrix over ranges suitable for field concentrations of florfenicol and florfenicol amine, showing good linearity during each day of testing (*R*^2^ always >0.99). Excellent accuracy and precision were demonstrated, for both analytes, by calculated bias always within ±15% and CV% always below 15% at all QC levels tested. The satisfactory outcomes obtained during recovery, matrix effect, and process efficiency investigations in serum and seminal plasma confirmed the strength of the method for the quantification of target compounds. To our knowledge, this is the first LC-MS/MS-validated approach for the quantification of florfenicol and florfenicol amine in serum and seminal plasma and was successfully applied for the determination of their concentration-time profiles in bulls. This paves the way to understanding the pharmacokinetics of this antibiotic and its active metabolite in bull's seminal plasma, which will enable the design of more appropriate treatment protocols.

## 1. Introduction

Florfenicol (FF) is a synthetic antibiotic belonging to amphenicol that acts as an inhibitor of protein synthesis through binding to the ribosomal subunits of bacteria. It shows a broad-spectrum activity against both Gram-negative and Gram-positive organisms, as well as mycoplasma and all organisms sensitive to chloramphenicol [1, 2]. FF was first approved for use in veterinary medicine in the European Union in 1995 as a feed or water additive for the treatment of bacterial infectious diseases in cattle, swine, chicken, dog, cat, and fish [2–4]. In particular, FF is commonly used in cases of bovine respiratory disease (BRD) in cattle, associated with *Mannheimia haemolytica, Pasteurella multocida,* and *Histophilus somni*. This antibiotic is also used to treat bovine interdigital phlegmon and bovine keratoconjunctivitis [2].

**Figure 1:**
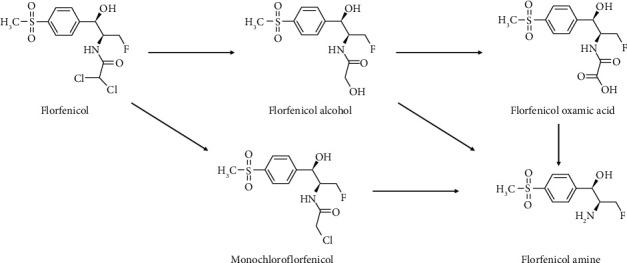
Florfenicol metabolic pathways.

Its lipophilicity allows FF to cross various anatomic barriers and achieve therapeutic concentrations against intracellular pathogens. For instance, in cattle, it can cross the blood-brain barrier up to 46% [5]. The half-life of FF in cattle is relatively short when administered intravenously (IV) but increases significantly after intramuscular (IM) and subcutaneous (SC) injection [2]. FF is primarily metabolized in the edible tissues of cattle, pig, chicken and fish, generating florfenicol amine (FFA). This happens through different bioconversion pathways, involving intermediate metabolites as florfenicol alcohol (FFOH), florfenicol oxamic acid (FCOOH), and monochloroflorfenicol (FFCl) [6, 7], as illustrated in Figure 1. Since FFA represents about 35% of the parent drug plasma concentration, it is considered the marker residue of FF by various international legislations, and maximum residual limits (MRLs) have been established for both compounds in all food producing animals [8].

In recent years, several analytical methods based on high-performance liquid chromatography (HPLC) [7, 9], high-performance liquid chromatography-tandem mass spectrometry (HPLC-MS/MS) [3, 10–13], gas chromatography (GC) [14], and gas chromatography-mass spectrometry (GC-MS) [15] have been reported for the determination of FF and its metabolites in water, feed, and animal-derived food. On the other hand, the analysis of animal biological fluids has been less frequent, mainly involving serum [16], plasma [13, 17], synovial fluid [18], and cerebrospinal fluid [19], but no study has focused on the pharmacokinetics of FF and FFA in seminal plasma. Since no information is available about their distribution in the genital tract, which would allow us to define the most correct treatment protocols, our research aimed to develop and validate a simple and quick approach to be applied to serum and seminal plasma samples collected during a pharmacokinetic study in bulls. As far as we know, a single method for FF and FFA quantification by LC-MS/MS in both matrices has never been proposed.

## 2. Materials and Methods

### 2.1. Chemicals and Reagents

Analytical standards of florfenicol (molecular weight: 358.21 g/mol; purity: 99.10%) and florfenicol amine (molecular weight: 247.29 g/mol; purity: 97.97%) were purchased from Dr. Ehrenstorfer (Augsburg, Germany); florfenicol-d3 (molecular weight: 361.23 g/mol; purity: 98.5%) was purchased from Toronto Research Chemicals (Toronto, Ontario, Canada). Acetonitrile, methanol, and ultra-pure water (all of LC-MS grade) were obtained from Merck (Milano, Italy). Before the start of the study, drug-free serum and seminal plasma samples were collected from healthy bulls and made available to the analytical laboratory for method development.

### 2.2. Standard Solutions

Stock solutions of FF and FFA at 1,000 *μ*g/mL were prepared by dissolving 10 mg of pure powder of each compound in a 10 mL volumetric flask containing methanol. Florfenicol-d3 (FF-d3) solution at 100 *μ*g/mL, which used as an internal standard (IS), was prepared by dissolving 1 mg of pure powder in a 10 mL volumetric flask containing methanol. Working solutions of florfenicol and florfenicol amine to be used for calibration and quality control (QC) samples were obtained by serial dilution of the stock solutions in acetonitrile and protected from light. All stock solutions were stored at −20 ± 2°C in the dark, and the stability of the three compounds over 1 month of storage was assessed.

### 2.3. Sample Preparation

All serum and seminal plasma samples were thawed at room temperature (20°C) and prepared using the technique described by Barbarossa et al. [20], with slight modifications. Briefly, 100 *µ*L of sample and 20 *μ*L of internal standard working solution (FF-d3 at 2 *μ*g/mL in acetonitrile) were transferred into a 1.5 mL Eppendorf microtube. Then, 80 *μ*L of acetonitrile was added, and protein precipitation was carried out by vortex mixing for 30 s and centrifuging at 21,000 ×g for 10 min at 20°C. Finally, 20 *μ*L of the supernatant was transferred into a LC glass vial containing 180 *μ*L of ultra-pure water.

### 2.4. Liquid Chromatography-Mass Spectrometry

Ultra-high-performance liquid chromatography (UHPLC) was performed on a Waters Acquity UPLC® system equipped with a binary pump, thermostated autosampler, column oven, vacuum degasser, and condenser (Waters, Milford, MA, USA). Chromatographic separation was obtained with a Waters Acquity BEH C18 (50 × 2.1 mm, 1.7 *µ*m) column coupled with the relative VanGuard precolumn (Waters, Milford, MA, USA) and maintained at 30°C. A gradient program was optimized using a mixture of ultra-pure water (A) and acetonitrile (B) at 0.3 mL/min, switching from 95 : 5 (*V*_*A*_ : *V*_*B*_) to 5 : 95 during the first 1.30 min, kept for 1.20 min, then back to 95 : 5 over 0.50 min, and finally re-equilibrated for 0.50 min before the following injection (total runtime 3.5 min). Samples were kept in the autosampler at 20°C, and 7.5 *μ*L from each vial was finally injected.

The detector was a Waters XEVO TQ-S microtriple quadrupole mass spectrometer (Waters, Milford, MA, USA), equipped with an electrospray ionization (ESI) source and operating in multiple reaction monitoring (MRM) mode. Capillary voltage was set at −2.80 kV for FF and FF-d3, and at +3.25 kV for FFA. Source and desolvation temperatures were 150 and 600°C, respectively. Cone gas was set at 50 L/h and desolvation gas at 900 L/h; argon was used as a collision gas. The analyte-dependent MS/MS parameters were optimized through combined infusion of a standard solution of each analyte and the LC mobile phase into the mass spectrometer. The most abundant transitions identified for FF, FFA, and FF-d3 are reported in Table 1 with their relative cone voltage and collision energy values.

Data acquisition and analysis were performed using MassLynx 4.2 software (Waters, Milford, MA, USA).

### 2.5. Validation

The technique was validated for each analyte following the European Medicines Agency ICH M10 guideline on bioanalytical method validation and study sample analysis [21] during three separated days of testing on serum and seminal plasma. The validation parameters considered included selectivity, calibration range, accuracy, precision (CV%), extraction recovery (RE), matrix effect (ME), process efficiency (PE), carry-over, stability, and reinjection reproducibility.

#### 2.5.1. Selectivity

After optimizing the chromatographic conditions, the retention time of FF, FFA, and FF-d3 was determined by injecting individual pure solutions at 0.01 *μ*g/mL. The selectivity of the method was assessed analysing ten blank bull serum and ten seminal plasma samples to verify the absence of chromatographic signals at the same elution time of FF, FFA, and FF-d3.

#### 2.5.2. Calibration Range

Matrix-matched calibration curves with a blank sample, a zero sample (blank sample spiked with IS), and calibrators at eight concentration levels were freshly prepared in both matrices in separate sessions following the procedure described in the sample preparation section (adding 20 *µ*L of FF or FFA spiking solution in acetonitrile, and then 60 *µ*L of acetonitrile). The calibration range (LLOQ-ULOQ) was 0.05–10 *μ*g/mL for FF in both serum and seminal plasma, 0.002–200 *μ*g/mL for FFA in serum, and 0.005–1000 *μ*g/mL for FFA in seminal plasma. The concentrations of all the calibrators for each curve are reported in Table 2. Peak area ratios between FF or FFA and the internal standard FF-d3 were plotted against their concentration, and a linear least squares regression model was applied. The accuracy of all the calibration standards should be within ±20% of the expected concentration at the LLOQ and below ±15% at all the other levels, and the resulting correlation coefficient (*R*^2^) was considered acceptable if ≥ 0.99. All calibrators had to produce chromatograms with a signal-to-noise (S/N) ratio >10.

#### 2.5.3. Accuracy and Precision

To evaluate the intra- and interday accuracy and precision of the method, quality control (QC) samples at four different concentrations (shown in Table 2) were prepared in 5 replicates along with each calibration curve. Accuracy, expressed as the relative difference between measured value and expected concentration, was evaluated at each QC level and considered acceptable if within ±15% the nominal concentration (±20% at the LLOQ). Similarly, precision, defined as the coefficient of variation (CV%) among repeated individual measures, had to be <15% (<20% at the LLOQ) for each QC level.

#### 2.5.4. Extraction Recovery, Matrix Effect, and Process Efficiency

The potential matrix effect was first verified by the postcolumn infusion technique: during the injection of a blank matrix samples in the LC-MS/MS system, standard solutions of each compound at 0.5 *µ*g/mL were coinfused in the MS interface to evaluate the stability of the produced signal.

An evaluation of RE, ME, and PE was performed following the approach described by Matuszewski et al. [22], in which peak areas obtained from three types of samples are compared: (A) Standard calibrators in mobile phase, containing the same amount of FF or FFA as the third QC level (1 *µ*g/mL of FF and 0.02 *µ*g/mL of FFA in serum; 1 *µ*g/mL of FF and 0.1 *µ*g/mL of FFA in seminal plasma); (B) blank samples of each matrix extracted as described above and added with the same amount of analyte; and (C) samples of each matrix fortified with the same amount of analyte and extracted as described above. Three replicates of each type of samples were prepared, using drug-free matrices collected from three different animals, in order to also assess possible subject-related differences. The following formulas were used to compare the three types of samples and evaluate RE, ME, and PE:

(1) $$\begin{array}{c} ME=\frac{B}{A}\left(\%\right)\text{,}\\ RE=\frac{C}{B}\left(\%\right)\text{,}\\ PE=\frac{C}{A}\left(\%\right)\text{.} \end{array}$$

#### 2.5.5. Carry-Over

For each matrix and analyte, blank samples were analysed immediately after the injection of the ULOQ (10 *μ*g/mL for FF in serum and seminal plasma; 0.2 *μ*g/mL for FFA in serum, and 1 *μ*g/mL for FFA in seminal plasma) to assess the absence of residual analyte.

#### 2.5.6. Stability and Reinjection Reproducibility

Different tests were performed to assess the stability of target analytes in the two matrices and in processed samples. The long-term stability of each analyte in serum and seminal plasma kept in the freezer (−20°C) was evaluated by preparing additional QCs (lowest and highest level, *n* = 3) to be analysed after 1 month of storage. The mean concentration at each level had to be within ±15% of the nominal concentration.

The stability of processed samples was first investigated by reinjecting the lowest and highest QCs (*n* = 5) from the first day of validation after being left in the autosampler (20°C) for 24 h. Similarly, QCs from the second and third days of validation were frozen (−20°C) after analysis, then thawed and reinjected after 48 h and 7 days, respectively, to assess reinjection reproducibility. For each series of samples, the mean concentration at each level had to be within ±15% of the nominal value.

The calculation of the arithmetic means of repeated samples and of the abovementioned validation parameters was performed using the Microsoft Excel software.

### 2.6. Application of the Method

The proposed method was developed to investigate the trends of florfenicol and florfenicol amine concentrations in bull serum and seminal plasma, providing novel information for optimized dosage regimens of this antibiotic. The suitability of this approach was assessed by analysing a preliminary series of samples collected at different timepoints (0, 12, 24, 36, 48, 72, 96, 120, 144, and 168 h) from a clinical and subclinical healthy bull (Hereford, 17 months, 331 kg) administered with FF at 20 mg/kg through IM injection in the neck. Semen was collected from the bull by electroejaculation using an electro-ejaculator in automatic mode with a two-electrode rectal probe of 60 mm diameter (Pulsator V, Lane Manufacturing, Denver, CO, USA). All the samples were immediately refrigerated at 4°C, then centrifuged for 30 min at 600 ×g and stored at −80°C within the first hour. The samples were transported to the UHPLC-MS/MS laboratory under controlled conditions, maintaining a temperature of −20°C throughout the shipment, and stored at −80°C upon arrival. Procedures used in the *in vivo* experiment were performed according to the standards for the “Use of Animals in Research and Education” by the World Organization for Animal Health (OIE) [23] and approved by the 3R Ranch owners.

## 3. Results and Discussion

### 3.1. Method Development

The method presented here was validated according to the current European Medicines Agency guidelines on bioanalytical method validation [21] and is capable of determining florfenicol and florfenicol amine concentrations in serum and seminal plasma. To the best of our knowledge, this is the first validated approach described for the quantification of FF and FFA in either biological fluid using UHPLC-MS/MS.

Different stationary phases (BEH C18 and HSS T3), mobile phase compositions (water and acetonitrile or methanol, with or without pH modifiers), and gradient eluent conditions were tested to optimize chromatography. The best resolution, peak shape, and intensity signal for FF, FFA, and FF-d3 was obtained on a BEH C18 column using a programmed combination of water and acetonitrile without any additive. These conditions resulted in optimal conditions also considering that florfenicol and florfenicol-d3 require negative electrospray ionization (ESI−), while florfenicol amine only produces detectable MS signals when positively charged (ESI+). With this setup, analysis can be carried out in a 3.5 min run, allowing the processing of even large batches of samples in a relatively short time.

A consistent part of the methods described in the literature employ organic solvents such as ethyl acetate [24, 25], acetone, dichloromethane [26], and acetonitrile with or without formic acid [27], or alkaline pH conditions for FF extraction from different animal tissues or feed. Other published applications for its quantification in plasma and serum are based on multiple liquid-liquid and/or solid-phase extraction techniques [16, 17]. We managed to avoid such expensive and time-consuming approaches, adapting a sample preparation procedure for tulathromycin quantification in plasma, seminal plasma, and urine previously validated by our group [20]. Moreover, compared to previous studies, the present method reduces sample and organic solvent volumes to just 100 *µ*L and bypasses the final filtration step. The lower amount of matrix required for analysis makes the approach suitable for PK studies, where repeated sample collection is necessary. The simple and quick sample treatment, consisting of protein precipitation and dilution of the sample, is a further strength point of this method, allowing it to process 24 samples in less than 15 min. Furthermore, FFA was not included in previous analytical applications on blood matrices [16–18]. In order to measure FF and FFA at levels comparable to those found in real serum and seminal plasma samples, different vial dilution factors were tested, and a twenty-fold dilution was finally chosen.

### 3.2. Method Validation

**Figure 2:**
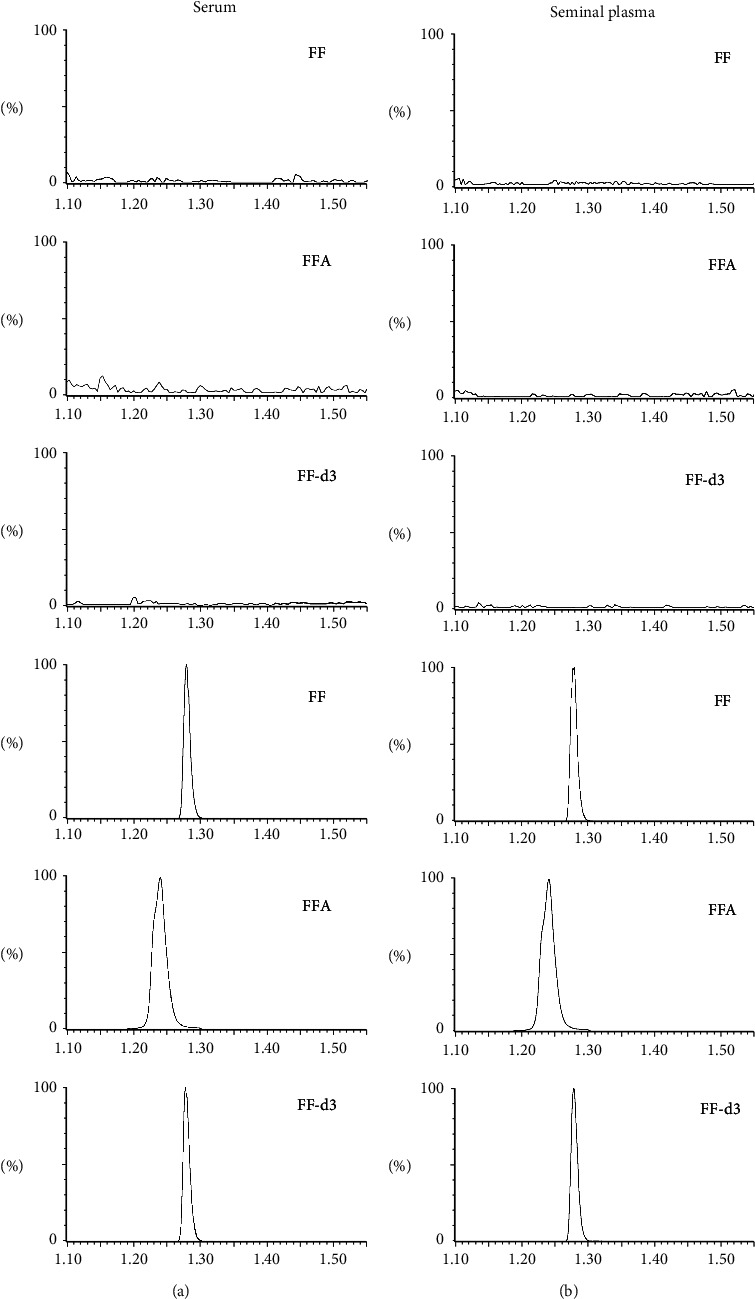
Chromatograms of the total ion current obtained for florfenicol (FF), florfenicol amine (FFA), and florfenicol-d3 (FF-d3) in serum (a) and seminal plasma (b), after injection of a blank sample (A) and of a sample at the LLOQ (B).

The injection of pure standards of florfenicol, florfenicol amine, and florfenicol-d3 allowed us to define their retention times, which were 1.28, 1.24, and 1.28 min, respectively. For each matrix and analyte, the analysis of ten blank samples did not show chromatographic interferences at the retention time of the monitored transitions, proving the good selectivity of the method, as shown in Figure 2.

In all the calibration curves analysed, the coefficient of determination (*R*^2^) was always ≥0.99, and calibration standards was always within ±15% of the nominal value which confirmed the linearity of the method over the specific ranges of concentration. Accuracy and precision were always within ±15% and <15%, respectively, at all QC levels in intraday and interday conditions (data are reported in Table 3).

**Figure 3:**
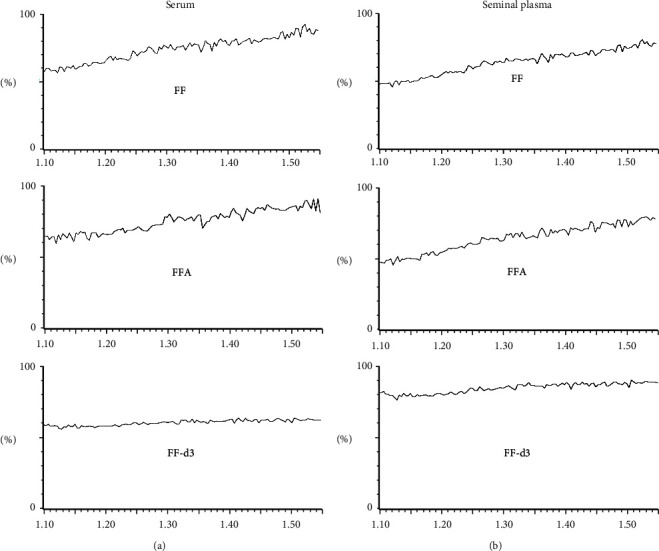
Assessment of the matrix effect through the acquisition of the signal obtained injecting a blank plasma of serum (a) or seminal plasma (b), while infusing florfenicol (FF), florfenicol amine (FFA), or florfenicol-d3 (FF-d3) standard solution at a constant flow rate.

During the postcolumn infusion test, no ionization suppression or enhancement were observed in the monitored transitions around the retention time of target analytes, giving a first demonstration of the absence of the matrix effect in both serum and seminal plasma. The chromatographic signals obtained for each analyte are shown in Figure 3. The analysis of standard calibrators in the mobile phase, blank matrix samples spiked before extraction, and blank matrix samples spiked after extraction confirmed the absence of any significant matrix effect, as well as the optimal recovery and global process efficiency. A complete summary of matrix effect (ME), recovery (RE), and process efficiency (PE) data is shown in Table 4. No carry-over was observed injecting blank samples following the highest point of each calibration curve.

The stability of target analytes in each matrix was assessed after 1 month at −20°C, and the average response remained always within ±8% of the initial value. Similarly, samples reinjected after being left for 24 h in the autosampler at 20°C or stored at −20°C for 48 h and 7 days did not show any relevant variation.

### 3.3. Application of the Method

**Figure 4:**
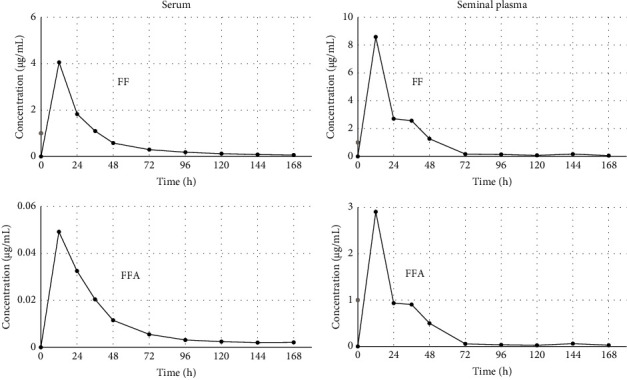
Concentration-time profile of florfenicol (FF) and florfenicol amine (FFA) in serum and seminal plasma, following intramuscular administration at 20 mg/kg in one bull.

The method was successfully applied to serum and seminal plasma samples collected during the evaluation of the pharmacokinetic profiles of FF and FFA in one healthy bull following IM administration of FF at 20 mg/kg. The obtained concentration vs. time curves are shown in Figure 4, proving that the measurement range of this approach is consistent with the levels of target analytes found in the two matrices. This investigation provided interesting information on the behaviour of this antibiotic and its active metabolite not just in serum but also in a novel matrix such as seminal plasma. The results showed higher levels of FF and FFA in seminal plasma than in serum and highlighted how they can both be found at relevant concentrations even 7 days after administration. Further confirmation of their long-lasting concentrations in seminal plasma will be critical to design appropriate treatment protocols (dose, route, and frequency), also in relation to specific MIC values associated with genital tract infections in bull.

## 4. Conclusions

The present work describes, for the first time, a single UHPLC-MS/MS technique for the quantification of florfenicol and its main metabolite, florfenicol amine, in bull serum and seminal plasma. The method combined an easy and fast laboratory procedure with optimal analytical performance. The validated ranges of concentrations were suitable for the detected levels of target analytes in each matrix and gave a first insight into their pharmacokinetics. The application of the technique to a larger number of patients will allow us to calculate the main PK parameters of FF and FFA and further investigate their behaviour in these biological fluids.

## Figures and Tables

**Table 1:** Selected mass transitions for florfenicol, florfenicol amine, and florfenicol-d3 and relative cone voltage and collision energy optimized values.

Analyte	Transitions monitored (*m/z*)	Cone voltage (V)	Collision energy (eV)
Florfenicol	**355.9** **⟶** **335.9**	40	8
	355.9 **⟶** 185.0	40	18

Florfenicol amine	**248.0** **⟶** **130.0**	40	21
	248.0 ⟶ 230.0	40	11

Florfenicol-d3	**359.0** ⟶ **187.9**	46	18
	359.0 ⟶ 338.9	46	8

Note: the product ion used for quantification is in bold.

**Table 2:** Calibrators and additional QC samples (*n* = 5, in bold) prepared for florfenicol and florfenicol amine in *serum* and *seminal plasma*.

Serum	Seminal plasma
*Florfenicol (μg/mL)*	
**0.05**	**0.05**
0.1	0.1
**0.25**	**0.25**
0.5	0.5
**1**	**1**
2.5	2.5
5	5
**10**	**10**

*Florfenicol amine (μg/mL)*	
**0.001**	**0.005**
0.002	0.01
**0.005**	**0.025**
0.01	0.05
**0.02**	**0.1**
0.05	0.25
0.1	0.5
**0.2**	**1**

**Table 3:** Intra- and interday accuracy and precision data obtained for FF and FFA in bull serum and seminal plasma at four different QC concentrations in five replicates (*n* = 5), during three separated days of validation.

	Serum			Seminal plasma		
	Accuracy (%)		Precision (%)	Accuracy (%)		Precision (%)
*Florfenicol*						
	QC1 (0.05 *μ*g/mL)			QC1 (0.05 *μ*g/mL)		
Day 1 (*n* = 5)	3.1		9.6	6.7		4.7
Day 2 (*n* = 5)	11.9		3.7	11.3		5.8
Day 3 (*n* = 5)	−1.6		5.3	4.0		8.4
Interday (*n* *=* 15)	4.4		8.1	7.3		6.3

	QC2 (0.25 *μ*g/mL)			QC2 (0.25 *μ*g/mL)		
Day 1 (*n* = 5)	0.4		2.6	2.2		2.5
Day 2 (*n* = 5)	5.7		0.4	2.9		4.7
Day 3 (*n* = 5)	1.7		3.0	−0.9		3.6
Interday (*n* *=* 15)	2.6		3.1	1.4		3.7

	QC3 (1 *μ*g/mL)			QC3 (1 *μ*g/mL)		
Day 1 (*n* = 5)	3.8		0.9	0.9		1.4
Day 2 (*n* = 5)	2.6		1.2	0.4		1.4
Day 3 (*n* = 5)	−0.8		4.2	−2.7		2.1
Interday (*n* *=* 15)	1.9		3.0	−0.5		2.3

	QC4 (10 *μ*g/mL)			QC4 (10 *μ*g/mL)		
Day 1 (*n* = 5)	2.2		2.4	3.0		3.2
Day 2 (*n* = 5)	2.9		1.3	3.5		2.0
Day 3 (*n* = 5)	4.6		0.5	2.5		1.9
*Interday (n* *=* *15)*	3.3		1.7	3.0		2.1

*Florfenicol amine*						
	QC1 (0.001 *μ*g/mL)			QC1 (0.005 *μ*g/mL)		
Day 1 (*n* = 5)	10.0		12.9	13.3		7.3
Day 2 (*n* = 5)	2.3		8.8	8.0		11.3
Day 3 (*n* = 5)	8.3		8.6	6.7		7.6
Interday (*n* *=* 15)	6.9		8.8	9.3		8.2

	QC2 (0.005 *μ*g/mL)			QC2 (0.025 *μ*g/mL)		
Day 1 (*n* = 5)	−8.7		1.9	−3.6		4.0
Day 2 (*n* = 5)	3.5		4.2	−0.3		5.1
Day 3 (*n* = 5)	−3.3		3.0	7.3		3.1
Interday (*n* *=* 15)	−2.9		6.0	1.2		6.0

	QC3 (0.02 *μ*g/mL)			QC3 (0.1 *μ*g/mL)		
Day 1 (*n* = 5)	−6.0		2.7	−5.7		3.0
Day 2 (*n* = 5)	1.0		3.1	−3.1		3.2
Day 3 (*n* = 5)	−5.3		3.2	−0.1		7.5
Interday (*n* *=* 15)	−3.4		4.3	−3.0		5.1

	QC4 (0.2 *μ*g/mL)			QC4 (1 *μ*g/mL)		
Day 1 (*n* = 5)	5.5		0.2	3.5		3.8
Day 2 (*n* = 5)	2.5		1.4	5.0		2.2
Day 3 (*n* = 5)	0.6		3.3	−3.5		3.2
Interday (*n* *=* 15)	2.9		2.5	1.7		4.7

**Table 4:** Results of matrix effect (ME), recovery (RE), and process efficiency (PE) experiments in the two matrices, obtained from three replicates of each type of sample at the QC3 spike level of FF or FFA.

	Mean peak area (arbitrary units, ×10^3^, *n* = 3)			ME (%)	RE (%)	PE (%)
	A	B	C			
*Florfenicol*						
Serum (1 *μ*g/mL)	216.4	207.1	193.5	95.7	93.4	89.4
Seminal plasma (1 *μ*g/mL)	186.9	172.3	164.6	92.2	95.5	88.0

*Florfenicol amine*						
Serum (0.02 *μ*g/mL)	11.4	10.8	9.6	94.7	88.6	84.2
Seminal plasma (0.1 *μ*g/mL)	34.3	32.6	29.4	95.0	90.3	85.7

Note: (A = standard calibrators in mobile phase, containing the same amount of analytes as the third QC level; B = blank samples of each matrix fortified after extraction with the same amount of analytes as the third QC level; C = samples of each matrix fortified with the same amount of analytes as the third QC level and extracted)

## Data Availability

The data used in this study are available upon reasonable request to the corresponding author.
